# Type I Interferon response in olfactory bulb, the site of tick-borne flavivirus accumulation, is primarily regulated by IPS-1

**DOI:** 10.1186/s12974-016-0487-9

**Published:** 2016-01-27

**Authors:** Chaitanya Kurhade, Loreen Zegenhagen, Elvira Weber, Sharmila Nair, Kristin Michaelsen-Preusse, Julia Spanier, Nelson O Gekara, Andrea Kröger, Anna K Överby

**Affiliations:** Virology, Department of Clinical Microbiology, Umeå University, Umeå, Sweden; Innate Immunity and Infection, Helmholtz Centre for Infection Research, Braunschweig, Germany; Department of Cellular Neurobiology, Technical University Braunschweig, Braunschweig, Germany; Institute for Experimental Infection Research, TWINCORE, Centre for Experimental and Clinical Infection Research, Hannover Medical School and Helmholtz Centre for Infection Research, Hannover, Germany; Department of Molecular Biology, Umeå University, Umeå, Sweden; Institute of Medical Microbiology, Otto-von-Guericke-University Magdeburg, Magdeburg, Germany; Present Address: Life and Medical Sciences Institute (LIMES), University of Bonn, Bonn, Germany; Present Address: Department of Pathology and Immunology, Washington University in St. Louis, St. Louis, MO USA

**Keywords:** Tick-borne encephalitis, IPS-1, Brain, Olfactory bulb, Type I interferons, Antiviral mechanism, Immune evasion

## Abstract

**Background:**

Although type I interferons (IFNs)—key effectors of antiviral innate immunity are known to be induced via different pattern recognition receptors (PRRs), the cellular source and the relative contribution of different PRRs in host protection against viral infection is often unclear. IPS-1 is a downstream adaptor for retinoid-inducible gene I (RIG-I)-like receptor signaling. In this study, we investigate the relative contribution of IPS-1 in the innate immune response in the different brain regions during infection with tick-borne encephalitis virus (TBEV), a flavivirus that causes a variety of severe symptoms like hemorrhagic fevers, encephalitis, and meningitis in the human host.

**Methods:**

IPS-1 knockout mice were infected with TBEV/Langat virus (LGTV), and viral burden in the peripheral and the central nervous systems, type I IFN induction, brain infiltrating cells, and inflammatory response was analyzed.

**Results:**

We show that IPS-1 is indispensable for controlling TBEV and LGTV infections in the peripheral and central nervous system. Our data indicate that IPS-1 regulates neuropathogenicity in mice. IFN response is differentially regulated in distinct regions of the central nervous system (CNS) influencing viral tropism, as LGTV replication was mainly restricted to olfactory bulb in wild-type (WT) mice. In contrast to the other brain regions, IFN upregulation in the olfactory bulb was dependent on IPS-1 signaling. IPS-1 regulates basal levels of antiviral interferon-stimulated genes (ISGs) like viperin and IRF-1 which contributes to the establishment of early viral replication which inhibits STAT1 activation. This diminishes the antiviral response even in the presence of high IFN-β levels. Consequently, the absence of IPS-1 causes uncontrolled virus replication, in turn resulting in apoptosis, activation of microglia and astrocytes, elevated proinflammatory response, and recruitment of inflammatory cells into the CNS.

**Conclusions:**

We show that LGTV replication is restricted to the olfactory bulb and that IPS-1 is a very important player in the olfactory bulb in shaping the innate immune response by inhibiting early viral replication and viral spread throughout the central nervous system. In the absence of IPS-1, higher viral replication leads to the evasion of antiviral response by inhibiting interferon signaling. Our data suggest that the local microenvironment of distinct brain regions is critical to determine virus permissiveness.

**Electronic supplementary material:**

The online version of this article (doi:10.1186/s12974-016-0487-9) contains supplementary material, which is available to authorized users.

## Background

The type I interferon system is the first line of defense against virus infections [1–5]. During viral replication, RNA intermediates are generated and recognized by distinct host pattern recognition receptors (PRRs). Two classes of PRRs, endosomal toll-like receptors (TLR) and cytoplasmic retinoid-inducible gene I (RIG-I)-like receptors (RLRs), are essential for the detection and protection against viral infection. However, the specific PRRs involved in mediating antiviral response are likely to be virus and cell type-specific [6–8]. Endosomal TLR3 and TLR7 recognize viral double-stranded RNA (dsRNA) and single-stranded DNA (ssRNA) and signal through their adaptor proteins TRIF (TLR3) and Myd88 (TLR7) to induce type I interferons (IFNs) via transcription factors IRF3 and IRF7, respectively [9]. RLRs such as RIG-I and melanoma differentiation antigen 5 (MDA5) are double stranded RNA binding DExD/H box RNA helicases [10, 11]. After the recognition of viral RNA, they interact with mitochondrial associated adaptor protein interferon -β promoter stimulator 1 (IPS-1; also known as MAVS, VISA, or CARDIF) [12–15]. This interaction leads to downstream signaling and activation of transcription factors IRF3 and NF-κB, which translocate to the nucleus and activate type I IFN and proinflammatory genes [16, 17].

Type I IFNs are locally produced in the central nervous system (CNS) upon viral infection. The producing cell types differ from parenchymal cells upon influenza and La Crosse virus to astrocytes upon vesicular stomatitis virus (VSV) infection [18–20]. However, very little is known about the importance and the relative contribution of the different PRRs in the different regions of the CNS for protection against neurotropic viruses.

Tick-borne encephalitis virus (TBEV) is one of the emerging arthropod-borne viral (arboviral) diseases in Europe and Asia. It is a neurotropic-enveloped RNA virus of the *Flaviviridae* family and closely related to other human pathogens such as yellow fever virus, dengue virus, West Nile virus (WNV), Japanese encephalitis virus (JEV), and Murray Valley encephalitis virus [21]. The virus is transmitted via tick bites or consumption of infected milk [22, 23]. The infection is characterized by a biphasic course in the primary phase, patients show signs of fatigue, headache, pain, and fever, followed by a second phase of neurological involvement with symptoms of encephalitis, meningitis, and paralysis. The mortality rate ranges from 0.5 to 30 %, with 30 to 60 % of survivors developing neurological sequelae [24–27]. Although an effective vaccine is available, treatment options are limited to supportive care [28]. Given such high medical importance along with the increasing number of cases, and expansion into new unaffected areas, many aspects of TBEV pathogenesis and immunity are still unclear. Langat virus (LGTV) is a naturally attenuated member of TBEV serogroup [29]. LGTV shares close molecular relationship (82–88 % amino acid identity) with TBEV and thus make it an ideal surrogate model to study TBEV pathogenesis [24].

We have recently shown that type I IFNs protect and control TBEV- and LGTV-induced inflammation and encephalitis by limiting systemic LGTV replication, spreading to CNS and the associated immunopathology [30]. However, the cell types and specific PRRs involved in IFN induction and clearance of LGTV are not known.

In the current study, we have determined the relative contribution of IPS-1 signaling in LGTV disease development and protection. We show that the tropism of the virus in the CNS is shaped by the IFN response, and that IPS-1 signaling is very important for IFN-β upregulation in the olfactory bulb. The absence of IPS-1 leads to uncontrolled viral replication in CNS but plays only a minor role in shaping the humoral immune response in periphery.

## Methods

### Ethics statement

All animal experiments were performed in compliance with the German animal welfare law (TierSchG BGBl. S. 1105; 25.05.1998). The mice were housed and handled in accordance with good animal practice as defined by FELASA. All animal experiments were approved by the responsible state office (Lower Saxony State Office of Consumer Protection and Food Safety) under permit number AZ 33.9-42502-04-11/0528.

### Mice and viral infections

C57BL/6 wild-type (WT) mice were purchased from Harlan. *IPS-1*^*−/−*^ mice on the C57BL/6 background were bred under specific pathogen free conditions at the Helmholtz Centre for Infection Research. LGTV strain TP21 and TBEV strain Hypr71 (G. Dobler) were propagated in Vero B4 cells. Titers were determined by focus forming assays on Vero B4 cells [31]. Six- to ten-week-old mice were intraperitoneally infected with the indicated focus forming units (FFU) of LGTV or TBEV in 100-μl phosphate buffered saline (PBS). For intracranial infections, mice were anesthetized by intraperitoneal injection with a mixture of ketamine (100 μg/g body weight) and xylazine (5 μg/g body weight). Mice were injected with indicated FFU of LGTV in 20 μl PBS. Mice that lost more than 20 % of their body weight were sacrificed and perfused with 10 ml of PBS. Experiments with TBEV strain Hypr were performed in the biosafety level 3 (BSL3) facility at the Helmholtz Center for Infection Research.

### Focus forming assay

Viral titers were determined by using a focus forming assay as described previously [31]. Briefly, serial dilution of virus samples were added on monolayer of Vero B4 cells in 96-well plates. After 1 h of incubation at 37 °C in a 5 % CO_2_, inoculum was removed and overlaid with 1.2 % Avicel RC-591 NF (FMC Biopolymer), 1× DMEM. Cell monolayers were fixed with 6 % paraformaldehyde dissolved in phosphate-buffered saline (PBS) 48 h post infection and permeabilized with 0.5 % triton X 100 and 20 mM glycine in PBS. LGTV foci were stained with monoclonal TBEV E antibody (monoclonal antiserum 1786 [32]) diluted 1:1000 in PBS supplemented with 10 % fetal calf serum (FCS) and 0.05 % tween 80 and horse reddish peroxidase-conjugated anti-mouse immunoglobulin (Thermo Scientific) diluted 1:2000 in PBS, 10 % FCS and 0.05 % tween 80. Antibody-bound infection foci were visualized with True Blue^TM^ Peroxidase substrate (KPL Inc., Maryland, USA), counted under microscope, and virus titer was expressed as focus forming unit per milliliter (FFU/ml).

### RNA extraction and real-time RT-PCR

RNA extraction from serum was done with QIAamp Viral RNA mini kit (QIAGEN), and LGTV levels were determine by comparing with a standard curve. The standard curve for viral RNA in serum was generated by RNA extraction, complementary DNA (cDNA) synthesis, and quantification with real-time reverse transcription PCR (RT-PCR) of control serum spiked with 1 – 10^5^ FFU of LGTV. Mice organs were homogenized in Trizol reagent (Invitrogen) using Lysis Matrix (Nordic Biolabs) and the tissue homogenizer Fast Prep-24 (MP). The RNA was extracted using the Nucleo-Spin RNA II kit (Macherey Nagel). Five hundred nanograms of total RNA was used to synthesize cDNA with the QuantiTect Reverse Transcription Kit (Qiagen). Expression levels of mouse GAPDH, IFN-β, IFN-α4, IFN-λ, Mx1, viperin, IL6, CCL5, CXCL10, IRF1, and TRIM79α were detected by validated QuantiTect primer assays (Qiagen) and the KAPA SYBR FAST quantitative polymerase chain reaction (qPCR) Kit using the 7900HT Fast-Real-time PCR System (Applied Biosystems). Viral RNAs were detected by two different TaqMan-based assays; the TaqMan probe for TBEV [33] detecting the 3′ NCR with a sensitivity of 10^4^ copies for LGTV or the newly developed LGTV NS3-based TaqMan assay, forward primer 5′-AACGGAGCCATAGCCAGTGA-3′, reverse primer 5′-AACCCGTCCCGCCACTC-3′ and probe FAM-AGAGACAGATCCCTGATGG-MGB, with a sensitivity of 10 copies. For both assays, the KAPA probe FAST qPCR kit was used. Signals of indicated messenger RNA (mRNA) or viral RNAs were normalized to the GAPDH mRNA signal.

### Brain immune cell quantification

Mice brains were harvested from uninfected or LGTV-infected mice. Following perfusion, brains were homogenized through a 70-μm cell strainer, digested with a collagenase solution (500 μg/ml collagenase D, 0.1 μg/ml trypsin inhibitor TLCK, 10 μg/ml DNase I, 10 mM HEPES in HBSS) for 1 h at room temperature. Cells were separated by centrifugation on a discontinuous 70-to-30 % Percoll gradient. For the detection of resident microglia, infiltrating macrophages and dendritic cells were incubated with anti-CD45, anti-CD11b, and anti-CD11c antibodies (BD Biosciences). Infiltrating T cells were detected using anti-CD3, anti-CD4, and anti-CD8 antibodies (BD Biosciences). Brain leukocyte numbers were quantitated using TruCount beads (BD Biosciences). Analysis was performed on BD LSRII using BD FACSDiva and FlowJo software.

### Immunhistology

Immunohistological analysis were performed from WT and *IPS-1*^*−/−*^ mice infected with LGTV at different time points (*n* = 3 per time point). Brains were removed after cardiac perfusion with PBS followed by 4 % paraformaldehyde (PFA) and incubated 24 h in 4 % PFA followed by an incubation in 30 % sucrose in 0.1 M phosphate buffer for an additional 24 h. Subsequently, the brains were frozen in Tissue Tek® Compound at -80 °C. 30 μm sagittal slices of the whole brain were cut using a freezing microtome (Frigomobil, Leica, Germany). All staining procedures were performed on free floating sections. Following a 1-h blocking step at room temperature in PBS containing 1 % BSA, 0.2 % Triton, and 10 % goat serum, the slices were incubated overnight at 4 °C in primary antibody solution consisting of 10 % goat serum and 0.2 % Triton in PBS. The following antibodies were used: monoclonal TBEV E antibody (monoclonal antiserum 1786 [32]) monoclonal mouse anti-GFAP (Sigma, 1:500), monoclonal guinea pig anti-NeuN (SySY, 1:500), polyclonal rabbit anti-IBA1 (Synaptic System, 1:500), and polyclonal rabbit anti- cleaved caspase 3 [Asp175] (Cell Signaling, 1:400). Secondary anti-mouse, anti-guinea pig, or anti-rabbit antibodies conjugated with FITC, Cy3, or Cy5 (Jackson ImmunoResearch) were incubated 1:500 in PBS for 2 h at room temperature. All analyzed samples were comparable and support the conclusion. Representative pictures are shown.

### Primary cell isolation and infection

Primary mouse hippocampal neurons were generated from WT and *IPS-1*^*−/−*^ mouse embryos E18 as described previously [34]. Briefly, 70,000 dissociated hippocampal cells were seeded on poly-l-lysine coated cover slips and incubated in Neurobasal medium (Invitrogen) supplemented with 2 % B27 (Invitrogen), 1× N-2 supplement (Invitrogen) and 0.5 mM Glutamax at 37 °C, 5 % CO_2_, and 95 % humidity. After 2 weeks, cells were infected with LGTV (MOI-0.001) and kinetics of viral replication was recorded by focus forming assay.

### Western blot analysis

For preparation of extracts, the brains or brain parts were lysed in buffer containing 250 mmol/L Tris, 0.5 % Triton X-100, and Halt protease inhibitor cocktail (Thermo Scientific, Schwerte, Germany). Western blot analysis was performed according to standard procedures. The following primary antibodies were used: Anti-STAT1-P (Tyr701, 58D6; Cell Signaling, Frankfurt, Germany), anti-STAT1 (STAT91/84; Transduction Laboratories Lexington, USA), and anti-actin (MAB 1501R, Chemicon, Limburg, Germany). Horseradish-peroxidase-conjugated anti-rabbit and anti-mouse antibodies (Amersham, Freiburg, Germany) were used as secondary antibodies using enhanced chemoluminescence detection (Bio-Rad, München, Germany). The chemiluminescence signal was recorded digitally by a ChemiDoc DRS imaging system (Bio-Rad, München, Germany). Digital signal acquisition and analysis were performed using the Quantity One Program (version 4.6; Bio-Rad, München, Germany).

## Results

### IPS-1-mediated type I IFN response controls LGTV replication in the CNS but has minor role in peripheral organs

**Fig. 1:**
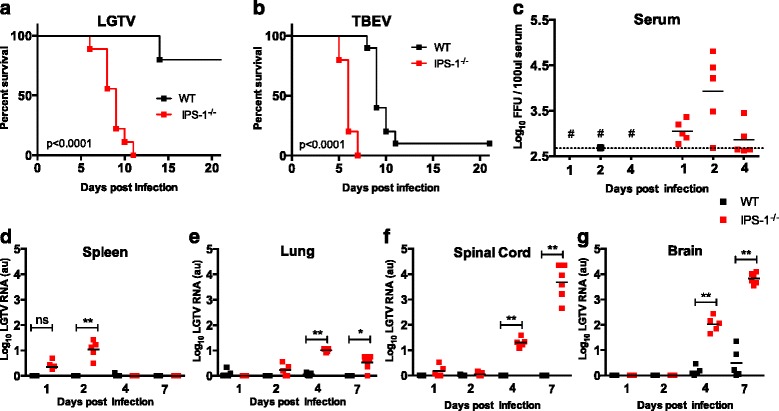
IPS-1 signaling controls LGTV replication in the CNS. **a**, **b** Survival of WT and *IPS-1* ^*−/−*^ mice. Mice were inoculated with 10^4^ FFU of LGTV (WT *n* = 10, *IPS-1* ^*−/−*^ *n* = 10) or TBEV strain Hypr 71 (WT *n* = 10, *IPS-1* ^*−/−*^ *n* = 5) via the intraperitoneal route and monitored for mortality for 21 days. Survival differences were tested for statistical significance by the log-rank test. **c** Viral burden in serum (*n* = 5). Serum were harvested various days post intraperitoneal infection of LGTV (10^4^ FFU). Viral RNA in the serum was quantified by real-time RT-PCR detecting the LGTV NS3 and compared with a standard curve derived from control serum samples spiked with LGTV. *Number sign* means not detectable; *dotted line* represents the detection limit. **d**–**g** Viral burden in different organs after intraperitoneal infection of LGTV (10^4^ FFU, *n* = 5–6) measured by real-time qPCR detecting LGTV NS3 gene (detection limit 10 copies) and normalized to intracellular GAPDH levels. **d** Spleen. **e** Lung. **f** Spinal cord. **g** Brain. *Asterisks* indicate statistical significance calculated by Mann-Whitney test, **p < 0.01, *p < 0.05. *au* arbitrary units

Previously, we showed that type I IFN response in both the peripheral and the central nervous system is indispensable for the control of LGTV and TBEV infection [30]. Here we were interested in understanding the PRR pathways responsible for antiviral innate immunity. To investigate the role of RIG-I-like receptors and wild-type (WT) and *IPS-1*^*−/−*^ mice were infected intraperitoneally with 10^4^ focus forming units (FFU) of LGTV or TBEV strain Hypr (Fig. 1a, b). *IPS-1*^*−/−*^ mice were found to be highly susceptible to LGTV infection, with a 100 % mortality rate, compared to WT mice that displayed only 20 % mortality (Fig. 1a). *IPS-1*^*−/−*^ mice started showing symptoms of paralysis, lethargy, hunchback posture, fur ruffling, and weight loss 2 days before death, with a median survival time of 9 days. *IPS-1*^*−/−*^ mice infected with highly pathogenic TBEV strain Hypr also showed 100 % mortality but died earlier with a median survival time of 6 days post infection (dpi) (Fig. 1b). This was in contrast to WT mice that exhibited longer survival; 90 % of WT mice died in a median time of 9 dpi, with encephalitic symptoms (*p* < 0.005). *IFNAR*^*−/−*^ mice, on the other hand, were more susceptible and succumbed within 5 dpi [30]. These data show that while IPS-1 signaling is essential for defense against LGTV and TBEV infection, other pattern recognition receptors also play an important role in protection against LGTV and TBEV infections.

We evaluated the viral burden in the serum, peripheral organs, and the CNS of LGTV-infected WT and *IPS-1*^*−/−*^ mice*.* Quantitative real time RT-PCR analysis of viral RNA detected higher LGTV levels in serum and only low levels of LGTV in peripheral organs of *IPS-1*^*−/−*^ but not in WT mice (Fig. 1c–e), indicating that higher viremia in serum of *IPS-1*^*−/−*^ mice results in higher viral load entering the brain. The most important LGTV was readily detected 4 dpi in the brain in both WT and IPS-1 mice, indicating that LGTV has a strong tropism of the brain (Fig. 1f–g).

Consistent with the importance of IPS-1 in type I IFN induction, ELISA analysis of serum from LGTV-infected mice revealed lower IFN-α levels in *IPS-1*^*−/−*^ mice (Additional file 1: Figure S1). These results indicate that IPS-1 mediated IFN response control systemic viral replication and higher viral replication in the circulation leads to a higher viral load in the brain. More importantly, IPS-1 is vital for controlling LGTV replication within the CNS.

### Enhanced inflammation and immune cell infiltration in the CNS of LGTV-infected *IPS-1*^*−/−*^ mice

**Fig. 2:**
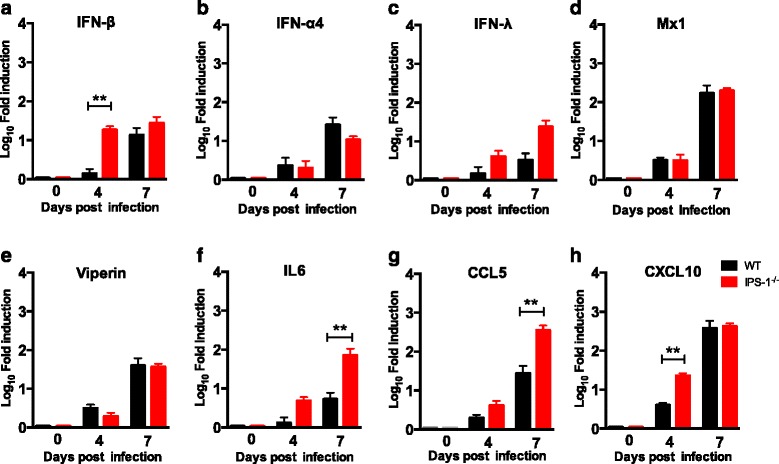
Impact of IPS signaling on chemokine, cytokine, and ISG expression upon virus infection. WT and *IPS-1* ^*−/−*^ mice were infected intraperitoneally with 10^4^ FFU of LGTV, and the brains were harvested on 0, 4, and 7 dpi. Expression levels of IFN-β, IFN-α4, IFN-λ, Mx1, viperin, IL6, CCL5, and CXCL10 were determined by real-time RT-PCR (**a**–**h**). Data represent the mean and standard error of the mean (SEM) of five to six mice per time point from at least two independent experiments. *Asterisks* indicates statistical significance calculated by Mann-Whitney test, **p < 0.01

Given the uncontrolled LGTV replication in CNS of *IPS-1*^*−/−*^ mice and the fact that such mice displayed encephalitic symptoms, next, we sought to understand how IPS-1 ablation influences virus-induced inflammation in the brain. RT-PCR analysis of brains 4 and 7 days post intraperitoneal LGTV infection revealed enhanced induction of IFN-β 4 dpi while no statistical difference in IFN-α4 and IFN-λ were observed (Fig. 2a–c). Mx-1 and viperin are key interferon inducible antiviral effectors. Curiously, in spite of a higher IFN-β response in *IPS-1*^*−/−*^ mice, due to a higher viral titer in such mice (Fig. 1g), no difference between WT and *IPS-1*^*−/−*^ was detected in the induction of Mx-1 and viperin (Fig. 2d, e). Importantly, activation of genes for proinflammatory cytokines such as IL-6 as well as those for various chemokines including CCL5 7 dpi and CXCL10 4 dpi was found to be significantly higher in brains of *IPS-1*^*−/−*^ mice (Fig. 2f–h). Thus, in the absence of IPS-1 signaling, LGTV infection induces a higher inflammatory response in the brain and a delayed interferon-stimulated gene (ISG) induction.

**Fig. 3:**
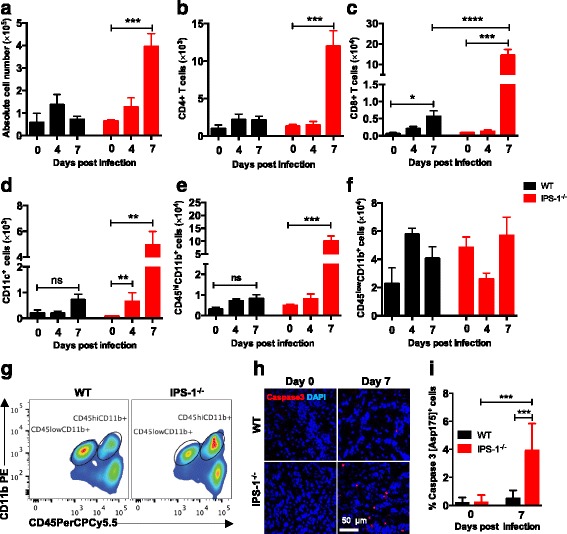
Increased CNS infiltration in the absence of IPS-1 signaling. WT and *IPS-1* ^*−/−*^ mice were infected intraperitoneally with 10^4^ FFU of LGTV. Leukocytes were isolated from the brains at different time points 0, 4, and 7 dpi by Percoll gradient centrifugation, stained for various cell markers, and analyzed by flow cytometry. **a** The total number of brain lymphocytes was determined by cell counting. **b** The total number of CD4^+^ T cells. **c** The total number of CD8^+^cells. **d** The total number of dendritic (CD11c^+^) cells. **e** The total number of infiltrating macrophages and neutrophils (CD45^hi^CD11b^+^). **f** The total number of microglia (CD45^low^CD11b^+^). **g** Representative flow cytometry diagram of CD45 and CD11b staining of brain leukocytes from WT and *IPS-1* ^*−/−*^ mice. Data represent the mean and standard error of the mean (SEM) of 3–10 mice per time point from at least two independent experiments. **h** WT and *IPS-1* ^*−/−*^ mice were infected intraperitoneally with 10^4^ FFU of LGTV, and mice brains were isolated for immunohistology (*n* = 5). Depicted are the immunohistological analyses of the olfactory bulb of uninfected and infected mice (7 dpi). Caspase 3 (*red*) and DAPI (*blue*). Magnification ×40, *scale bar* 50 μm. **i** Quantification of Caspase 3 (Asp175^+^)-positive cells in the olfactory bulb of uninfected and infected mice (7 dpi). *Asterisks* indicates statistical significance calculated by Mann-Whitney test, ****p < 0.0001, ***p < 0.001, **p < 0.01, *p < 0.05. *ns* not significant

Next, we employed flow cytometry to evaluate the effect of IPS-1 deficiency on the recruitment of inflammatory cells into the brain. *IPS-1*^*−/−*^ mice exhibited brain-infiltration by different immune cells types including macrophages, neutrophils (CD45^hi^CD11b^+^), DC (CD11c^+^), and T cells (CD4^+^ and CD8^+^) (Fig. 3a–e). The immune cell infiltration was detectable 7 dpi and was associated with leakage of the blood brain barrier, as revealed by Evans blue injection (Additional file 2: Figure S2) and not at earlier time points such as 4 dpi. Notably, a slight increase in the DC population was however observed on 4 dpi in brains of *IPS-1*^−/−^ mice when the BBB was intact (Fig. 3d). This observation could be due to differentiation of microglia into brain resident DCs as a result of production of proinflammatory cytokines [35]. This is in contrast to brain-resident microglia (CD45^low^CD11b^+^) that remained unchanged on 7 dpi (Fig. 3f). Direct killing of the neurons by apoptosis has been reported for both LGTV and TBEV in cell culture [36, 37], and apoptosis in the brain might influence the susceptibility of the mice to LGTV infection. We therefore set out to investigate the levels of apoptosis in WT and *IPS-1*^*−/−*^ brains after LGTV infection. We employed immunohistochemistry to determine the extent of apoptosis by analyzing cleaved caspase 3 (Asp175) levels in LGTV-infected WT and *IPS-1*^*−/−*^ mice brain. High level of apoptosis of brain cells was observed especially in the olfactory bulb of *IPS-1*^*−/−*^ mice on 7 dpi while only few apoptotic cells can be seen in the same region of WT mice (Fig. 3h, i).

We did not observe any difference in immune cell number or composition of spleen in LGTV-infected WT and *IPS-1*^*−/−*^ mice (Additional file 3: Figure S3. 2A–I). In addition, the neutralizing antibody response was also not changed (Additional file 3: Figure S3 2J. Additional file 4: Method S1). In overall, these data suggest that in the absence of IPS-1 signaling, LGTV replicate to higher levels in the CNS, leading to the release of inflammatory cytokines, which in turn results in a massive infiltration of immune cells to the site of inflammation and apoptosis in the brain.

### IPS-1 signaling is vital for restricting LGTV replication in neurons

**Fig. 4:**
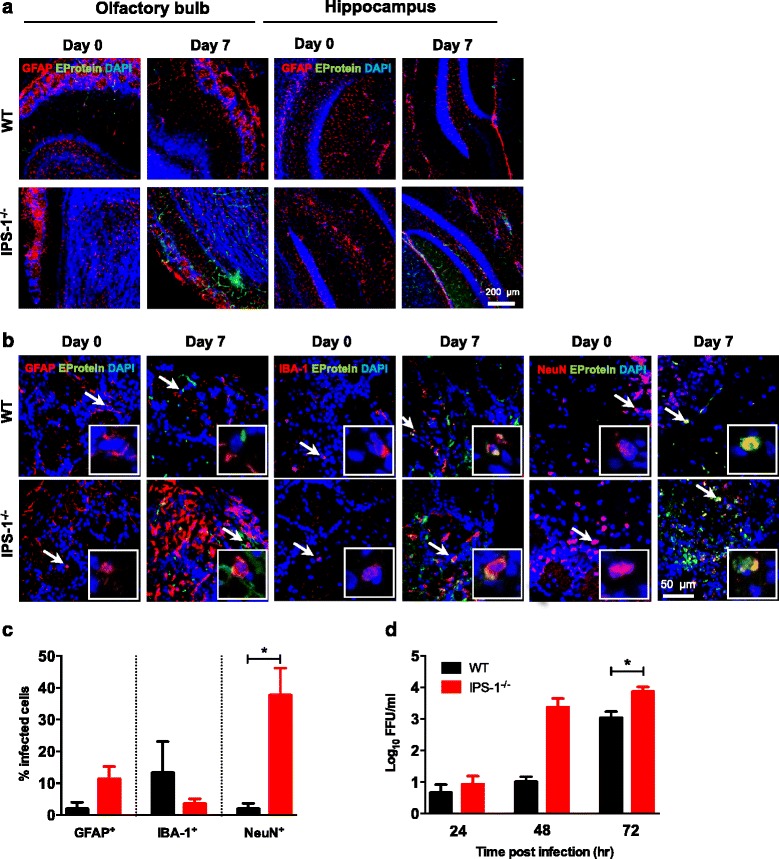
IPS-1 is important for controlling infection in the CNS. WT and *IPS-1* ^*−/−*^ mice were infected intraperitoneally with 10^4^ FFU of LGTV, and mice brains were isolated for immunohistology. **a** Depicted are the immunohistological analyses of the glomerular layer of the olfactory bulb and hippocampus of uninfected and infected mice (7 dpi). Representative pictures of at least two mice per group, GFAP (*red*), LGTV E-protein (green), and DAPI (*blue*). Magnification ×10, *scale bar* 200 μm. **b** Depicted are the immunohistological analyses of the glomerular layer of the olfactory bulb in uninfected and infected mice (7 dpi). Representative pictures of at least two mice per group, GFAP or IBA-1 or NeuN (*red*), LGTV E-protein (*green*), and DAPI (*blue*). Virus-infected astrocytes, microglial cell, and neurons are indicated by a *white arrow*. Magnification ×40, *scale bar* 50 μm. **c** Quantification of LGTV positive GFAP, IBA-1, and NeuN-positive cells in glomerular layer of the olfactory bulb in infected mice (7 dpi). **d** Primary neurons isolated from hippocampus of WT and *IPS-1* ^*−/−*^ were infected with 0.001 MOI of LGTV; viral burden were analyzed by focus forming assay 24, 48, and 72 h post infection. Data represent the mean and standard error of the mean (SEM) of from at least three independent experiments. Asterisks indicate statistical significance calculated by unpaired *T* test * *p* < 0.05

As described above, upon intraperitoneal infection, LGTV is able to spread from the periphery into the CNS. To investigate the impact of IPS-1 on viral replication and spread, we employed immunohistochemistry to determine the anatomical location of the virus in the brain (Fig. 4a). Viral E protein was detectable in the olfactory bulb and the hippocampus on 7 dpi in both WT and *IPS-1*^*−/−*^ mice. However, increased numbers of infected cells were detected in *IPS-1*^*−/−*^ mice. Viral E protein was mainly observed in the olfactory bulb in neurons (Fig. 4b). However, some of the neurons in the glomerular layer of the olfactory bulb are not terminally differentiated to express NeuN [38], so not all LGTV-infected cells could be identified with the NeuN marker. Further analysis revealed an activated phenotype of the majority of astrocytes (GFAP^+^) and microglia (IBA1^+^) (Fig. 4b, c). The percentage of infected cells in glomerular layer of the olfactory bulb was quantified, and we could detect a strong increase in infected neurons of *IPS-1*^*−/−*^ mice compared to WT. We also detected an increased susceptibility of astrocytes and a decreased susceptibility of microglial cell in *IPS-1*^*−/−*^ mice (Fig. 4c). These data indicate that mainly the neuronal cells are infected by LGTV. To further define the importance of IPS-1 signaling in neurons, primary embryonic hippocampal neurons were isolated and infected with LGTV MOI 0.001, viral growth was assessed by focus forming assay. The viral titers were higher in IPS-1 deficient neurons compared to WT (Fig. 4d). These results indicate that LGTV mainly targets neurons and that IPS-1 signaling in these cells is vital for controlling viral replication.

### Local IPS-1 signaling within CNS is important for protection against LGTV infection

**Fig. 5:**
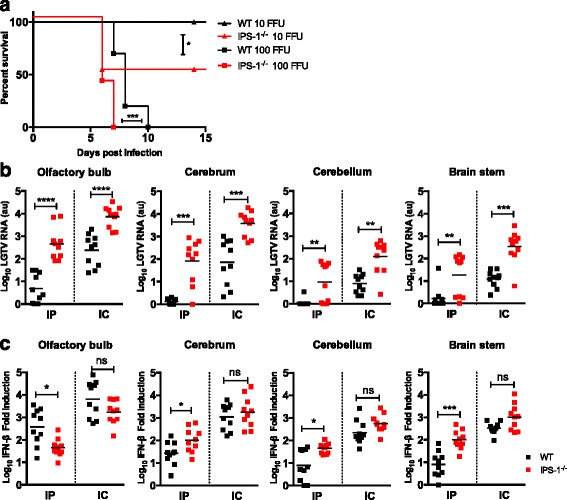
IPS-1 signaling controls local antiviral response within CNS. **a** Survival analysis of WT and *IPS-1* ^*−/−*^ mice. Mice (*n* = 9–10) were inoculated with 10 or 100 FFU of LGTV via the intracranial route and monitored for mortality for 21 days. Survival differences were tested for statistical significance by the log-rank test, * *p* < 0.05. **b**–**c** WT and *IPS-1* ^*−/−*^ mice were infected with LGTV intraperitoneally (10^4^ FFU) or intracranially (10^2^ FFU) and different regions of the brain, olfactory bulb, cerebrum, cerebellum and brain stem, were harvested 7 dpi and 5 dpi, respectively. Viral burden (**b**) were quantitated by real-time qPCR detecting the 3′ NCR (detection limit 10^4^ copies) *n* = 10 per experiment. IFN-β fold induction (**c**) after infection was calculated by measuring IFN-β levels in uninfected and infected brain parts by real-time RT-PCR, setting the IFN-β level in uninfected control samples to one and calculate the fold induction of IFN-β RNA transcript from infected brain parts. *Asterisks* indicates statistical significance calculated by Mann-Whitney test, ****p < 0.0001, ***p < 0.001, **p < 0.01, *p < 0.05. *ns* not significant, *au* arbitrary units

The above data indicate that after peripheral infection, virus invades the CNS and replicates more efficiently in *IPS-1*^−/−^ mice brains. However, to specifically understand the local role of IPS-1 signaling in the protection of the CNS against LGTV infection, we directly injected virus intracranially in WT and *IPS-1*^−/−^ mice (Fig. 5a). Although both WT and *IPS-1*^*−/−*^ mice succumbed to 100 FFU of LGTV, *IPS-1*^−/−^ mice died earlier, within 7 days compared 10 days for WT. However, a greater difference between WT and *IPS-1*^-/-^ was detected when challenged with a low dose of LGTV (10 FFU). All WT but only 50 % of *IPS-1*^−/−^ mice survived compared to the *IFNAR*^−/−^ mice that died [30]. This indicates that IPS-1 is very important for optimal antiviral response of the CNS, but other IPS-1 independent pathways must also be involved in the control at low viral infection doses.

### Different brain regions show distinct dependency on IPS-1 signaling for IFN-β production

Type I IFN response varies in neurons isolated from different brain parts [39]. To determine if IPS-1 signaling preferentially protects a specific brain region from LGTV infection, the brain parts were analyzed for virus replication (Fig. 5b). WT and *IPS-1*^*−/−*^ mice were infected with LGTV intraperitoneally (10^4^ FFU) or intracranially (10^2^ FFU), and brain regions were harvested 4 and 7 dpi for intraperitoneal and 5 dpi for intracranial infection. Upon peripheral administration of LGTV, viral replication was restricted to the olfactory bulb in WT mice (Fig. 5b). High levels of viral replication was observed already 4 dpi in olfactory bulb in *IPS-1*^*−/−*^ mice compared to the other brain regions (Additional file 5: Figure S4), indicating that LGTV preferentially targets the olfactory bulb. However, in the absence of *IPS-1*^*−/−*^, the infection was established earlier in the brain compared to WT. Upon 7 dpi LGTV spread to cerebrum however, the RNA levels in cerebellum and brain stem remained low. For intracranial injection, a similar trend was observed; however, the LGTV RNA levels were higher in *IPS-1*^*−/−*^ in all brain regions compared to WT (Fig. 5b).

Since IPS-1 signaling is important for the production of type I IFN, we analyzed mRNA levels of IFN-β in the different brain regions (Fig. 5c). Surprisingly, IFN-β mRNA levels in the cerebrum, cerebellum, and brain stem of *IPS-1*^*−/−*^ mice were higher or comparable to those of WT mice, indicating that IPS-1 plays only a minor role in the induction of IFN-β in these brain regions. In contrast, upon intraperitoneal infection, lower mRNA level of IFN-β (*p* = 0.0115) was present in the olfactory bulb of *IPS-1*^*−/−*^ mice compared to WT mice. Although exhibiting a higher viral titer in all brain parts, intracranially infected animals display IFN-β levels comparable to that in WT mice, indicating that IPS-1-mediated type I IFN production is limited to the olfactory bulb.

### Specific role of IPS-1 signaling in antiviral response in the olfactory bulb

**Fig. 6:**
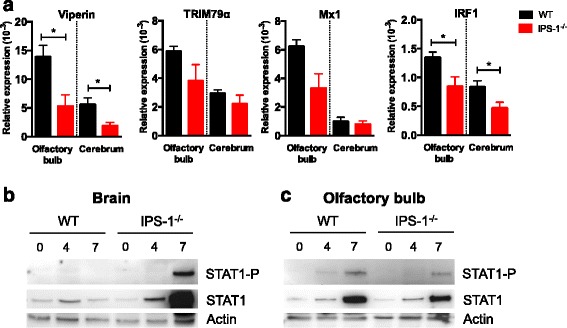
*IPS-1* ^−/−^ mice show lower basal expression of antiviral ISGs and a delayed STAT1 phosphorylation in the olfactory bulb after LGTV infection. **a** Basal expression level of viperin, TRIM79α, Mx1, and IRF1 were determined by real-time RT-PCR for the olfactory bulb and cerebrum of uninfected WT and *IPS-1* ^-/-^ mice. The data is displayed as relative expression levels values compared to the basal level of GAPDH. Data represent the mean and standard error of the mean (SEM) of five mice. *Asterisks* indicates statistical significance calculated by Mann-Whitney test, *p < 0.05. WT and *IPS-1* ^*−/−*^ mice were infected with LGTV intraperitoneally (10^4^ FFU), and whole brain (**b**) and olfactory bulb (**c**) were dissected out 0, 4, and 7 dpi and protein analysis of the STAT1-P, STAT1, and actin was measured by Western blot. Representative pictures are shown (*n* = 2)

The data obtained thus far suggested that LGTV establishes its infection first in the olfactory bulb and the IFN-β response induced by LGTV infection is not sufficient to control viral replication in the absence of IPS-1. To address the role of ISG expression during early stages of infection, we analyzed basal expression of TBEV/LGTV-specific antiviral ISGs [40–42] in the cerebrum and olfactory bulb. Basal expression levels of viperin and IRF1 were significantly lower in the olfactory bulb and cerebrum of *IPS-1*^*−/−*^ mice (Fig. 6a), while similar levels of TRIM79α and Mx1 were present (Fig. 6a). A lower base line expression of some antiviral ISGs could increase the susceptibility of IPS-1-deficient mice, leading to higher viral replication and higher levels of the interferon signaling antagonist NS5. LGTV NS5 protein has previously been shown to inhibit STAT1 phosphorylation and upregulation of ISGs in vitro [43, 44]. To test this hypothesis, that high viral replication results in low STAT1 phosphorylation, STAT1 phosphorylation in the whole brain and different brain regions of LGTV-infected WT and *IPS-1*^*−/−*^ mice was analyzed. Phospho-STAT1 was only detected 7 dpi in *IPS-1*^*−/−*^ whole brain even though viral replication, and high IFN-β were detected at day 4 in such mice (Figs. 1g , 2a, and 6b). When analyzing STAT1-P in the different brain parts separately, a clear signal was detected 4 and 7 dpi in olfactory bulb of WT mice. A similar signal was detected in *IPS-1*^*−/−*^ mice only 7 dpi (Fig. 6c). These results suggest that the increased susceptibility of *IPS-1*^*−/−*^ mice to LGTV infection depends on a complex interplay between many factors. First, the decreased baseline levels of antiviral ISGs in the CNS give the virus an advantage in *IPS-1*^*−/−*^ mice. This is further exacerbated by the fact that LGTV-induced IFN-β response in the olfactory bulb is IPS-1-dependent. Finally, even though viral replication leads to IFN-β upregulation, the increase in viral IFN signaling antagonist inhibits the STAT1-P at early time points thereby decreasing the viperin and Mx1 response in the CNS at that point (Figs. 1g, 2a, d–e, and 6c).

## Discussion

Protection against neurotropic LGTV infection requires coordinated action of the type I IFN system in both peripheral and CNS to prevent LGTV-induced inflammation and development of encephalitis [30]. Here, we have characterized the function of IPS-1, a key adaptor molecule that act downstream of RLRs (RIG-I and MDA5) to activate the IFN and NF-κB pathways in LGTV infection. We have shown that IPS-1 signaling is important for controlling lethal LGTV infection. IPS-1-deficient mice show high viral replication in the peripheral and CNS, increased BBB permeability, massive infiltration of immune cells, and uncontrolled inflammation. After close analysis of different brain sections, we found that IPS-1 signaling plays a role in determining the base line levels of some antiviral ISGs but also controlling LGTV viral replication in all brain sections. Upregulation of IFN-β is specifically dependent on IPS-1 in olfactory bulb. Thus, we propose a specific role of pattern recognition receptors in the different sections of the brain in neurotropic LGTV infection.

Neuroinvasiveness and neurovirulence are key steps in the pathogenesis of neurotropic viruses. The type I IFN system is an important component of innate immunity and limits viral load of many flavivirus infections [4]. Previously, we showed that *IFNAR*^*−/−*^ mice succumb to LGTV infection within 5 days with uncontrolled systemic viremia [30]. LGTV-infected *IPS-1*^*−/−*^ mice showed declined systemic type I IFN responses. This finding agrees with our previous studies indicating that recognition of LGTV is IPS-1-dependent [31] and that induction of type I IFN production by other RNA viruses is mediated by IPS-1-dependent mechanisms [45–47]. The lower systemic IFN response probably contributed to higher systemic viral levels, thus contributing to higher viral titers and earlier time point of neuroinvasiveness (Additional file 5: Figure S4 ). However, no difference in neutralizing antibody titers or spleen immune cell composition was detected. These later findings are markedly different compared to the effect seen in WNV infected *IPS-1*^*−/−*^ mice [47]. Virus entry into the brain by LGTV, TBEV, and Japanese encephalitis is independent of the integrity of the BBB because virus is detectable even when the BBB is intact [30, 48, 49], and this is also true in IPS-1 deficient-mice.

The brain has distinct immune responses to pathogens and injury where resident brain cells including microglia and astrocytes, which are unique innate immune cells without direct counterparts in the periphery, can produce IFN and proinflammatory cytokines and can crosstalk with infiltrating immune cells [50–53]. Local type I IFN response is very important for controlling lethal LGTV infection in the CNS [30]. Indeed, high levels of LGTV replication were detected in the brain of IPS-1-deficient mice compared to WT. This in turn could lead to elevated IFN-β response in the brains due to increased activation of IPS-1-independent pathways. However, increased IFN response neither led to the induction of antiviral effector genes early in infection nor was it able to control the viral infection. One explanation might be that LGTV and TBEV non-structural protein NS5 can inhibit JAK STAT pathway downstream of IFNAR to block effector functions of IFN, e.g., upregulation of ISGs [43, 44]. Accordingly, in spite of the presence of high levels of viral RNA and IFN-β in brains of *IPS-1*^*−/−*^ mice, day 4 post infection, STAT1 phosphorylation was not detectable and neither was ISG expression. Other studies have shown that even in the presence of type I IFN, viral replication could not be controlled and that other mechanisms like IRF-1 are essential to control viral replication [34]. IPS-1 located on peroxisomes has been shown to induce an IRF-1-dependent IFN-independent antiviral mechanism [54], which has been shown to be important for both VSV and HCV [34, 55]. This pathway might be one of the reasons why IPS-1-deficient mice cannot control the LGTV infection in CNS.

Higher viral burden observed in *IPS-1*^*−/−*^ mice brains was a result of higher viral replication in neurons, similar to LGTV-infected *TLR7*^*−/−*^ mice brains [56]. Although not highly infected, astrocytes and microglia showed an activated phenotype as evident from increased expression of GFAP and IBA-1, respectively. Astrocytes are the most abundant glial cell population in the brain and when activated could act as potential source of proinflammatory cytokines. These cytokines might contribute to BBB breakdown and TBEV induced neurotoxicity and thus play a major role in TBEV pathogenesis [57, 58]. We also detected a strong inflammatory response in the *IPS-1*^*−/−*^ mice leading to the opening of BBB and activation of the cell-mediated immune response with high quantity of infiltrating CD4 and CD8 T cells in later stages of infection. Although a strong T cell response was observed, it was not protective; similar findings were also seen in case of WNV [47]. Notably rather than protection, this higher T cell response in the brain contributed to immunopathology [59]. Apoptosis in the brains of LGTV-infected *IPS-1*^*−/−*^ mice were apparent as a result of high viral infection and inflammatory response, which combined ultimately lead to the death of mice.

Local immune response within the CNS plays an important role in combating viral infections [60]. In the absence of IFNAR signaling in the CNS, LGTV replicated to higher levels and mice succumbed early to the infection [30]. Studies with Sindbis, JEV, and WNV reports that the IPS-1 pathway is important in controlling viral replication in the brain [47, 61, 62]. Comparison of the different brain regions displays specific antiviral mechanisms [39, 63, 64]. Two neuronal subtypes, granule cell neurons of the cerebellum and cortical neurons from the cerebral cortex, have unique innate immune programs and showed differential permissiveness to replication of several positive-stranded RNA viruses [39]. Cerebellum has also been shown to be more resistant to WNV viral infection compared to cerebrum [39], and this holds true for LGTV as well. One explanation for this might be an increased basal expression of ISGs such as Ifi27, Irg1, viperin, and Stat1 in granule neurons [39]. IPS-1 signaling seems to be very important to restrict LGTV growth throughout the brain. Although, high IFN-β levels were detected in *IPS-1*^*−/−*^, it could not restrict replication in any of the brain regions. Comparison of the basal expression levels of some ISGs known to be active against TBEV, LGTV, or flaviviruses in general [41, 42, 65, 66] revealed that the olfactory bulb generally shows higher levels of viperin, TRIM79α, Mx1, and IRF1 compared to cerebrum. This might play a role for defense against pathogens which enter the brain preferentially via the olfactory route. IPS-1-deficient mice have lower expression of some specific ISG but not all. Since TBEV and LGTV are very sensitive to the antiviral action of viperin [42] (unpublished data), this might be one of the contributing reasons why IPS-1-deficient mice are more susceptible to LGTV infection. This initial advantage might also contribute to higher levels of viral NS5 and inhibition of STAT1 phosphorylation in IPS-1 deficient mice. This is in contrast to wild-type mice that have higher basal levels of antiviral ISGs, respond faster by production of IFN-β, and have a more robust STAT1-mediated expression of ISGs required to contain the infection. Notably, LGTV infection was restricted to olfactory bulb in WT mice. Similar observations were made in VSV and cytomegalovirus (CMV) infections. VSV- and CMV-induced long-distance interferon signaling within the brain that blocks virus spread [67] and astrocytes were found to be the main producer of IFN-β in the olfactory bulb in response to VSV infection [18]. In LGTV infection, the olfactory bulb seems to be more dependent on IPS-1 signaling for IFN-β production compared to the other brain parts, where RLR independent pathways (MyD88 and TRIF) compensated with higher IFN-β production. Thus, different cell types might exert specific immune response in different sections of the brain.

## Conclusions

In summary, IPS-1 signaling is important to coordinate and shape the innate immune response within the peripheral and CNS during LGTV infection. In our study, we show that some antiviral ISGs have lower basal expression levels in IPS-1 deficient mice, which may contribute to a higher viral burden. We also show that IPS-1 signaling is more important for IFN-β production in olfactory bulb compared to the other brain regions. We propose that various PRR pathways regulate the IFN response within different sections of the brain. To this end, further studies are underway to determine what cell types and antiviral factors govern the differential role of PRRs in the different regions of the brain during LGTV and TBEV infections. This study will contribute to the understanding of regional immunological diversity within the CNS and its impact on neurotropic virus infections.

## Additional files

Additional file 1: Figure S1.
*IPS-1* ^*−/−*^ mice have lower IFN-α levels in serum after LGTV infection compared to WT mice. WT and *IPS-1* ^*−/−*^ mice were infected intraperitoneally with 10^4^ FFU of LGTV, and serum samples were collected at indicated time points (*n* = 5–10). The amount of IFN-α in the mouse serum was determined by enzyme linked immunosorbent assay (ELISA) according to the manufacturer’s instructions (PBL). Significance was calculated with student’s *T* test, **p* < 0.05. (PDF 53.6 KB) Additional file 2: Figure S2. Effect of IPS-1 signaling on BBB permeability upon LGTV infection. WT and *IPS-1* ^*−/−*^ mice were mock or intraperitoneally infected with 10^4^ FFU of LGTV (*n* = 3). Mice were intravenously injected with 100 μl 2 % Evans blue (Sigma) in PBS 4 and 7 dpi. After 1 h, animals were transcardially perfused with 20 ml PBS and the brains were removed. The brains were weighted and homogenized in 50 % TCA, and absorbance was measured at 610 nm. The absorbance was divided by the weight of the sample and normalized to mock infected samples. (PDF 42.5 KB) Additional file 3: Figure S3. IPS-1 does not influence the humoral response or cell composition or number in the spleen. WT and *IPS-1* ^*−/−*^ mice were infected intraperitoneally with 10^4^ FFU of LGTV. Spleens were harvested 4 and 7 dpi, and immune cells were isolated, counted, stained for various cell markers and analyzed by flow cytometry (*n* = 5–10). (A) Absolute cell numbers. (B) Total CD4^+^ T cells. (C) Total CD8^+^ cells. (D) Total B220^+^ cells. (E) Total CD11b^+^ cells. (F) Total CD11c^+^ cells. (G) Total NKT cells. (H) Total NK cells. (I) Total regulatory T (Treg) cells. (J) WT and *IPS-1* ^*−/−*^ mice were mock or intraperitoneally infected with 10^4^ FFU of LGTV, and the serum was isolated on 0, 4, and 7 dpi. Neutralizing antibody titers were determined by virus neutralization assay. Data represents mean with SEM of 5–10 mice in each group per time point. (PDF 20.6 KB) Additional file 4:
**Method S1.** Virus neutralization assay. Neutralization titer of antibody solution was determined by modified method of plaque reduction neutralization test (PRNT) called as rapid fluorescent focus inhibition test (RFFIT) [68, 69]. Briefly, mice sera were diluted 1:10 in serum-free maintenance media and heat-inactivated. The sera were serially diluted and incubated with 50 FFU LGTV for 1 h, and focus forming assay was performed. Neutralizing antibody titer was calculated as the reciprocal of serum dilution that gave 50 % (NT_50_) reduction of the number of FFU as compared to virus control. The test was accepted if virus dose was in the range of 30–90 FFU. (DOC 21.5 KB) Additional file 5: Figure S4. Enhanced viral replication in olfactory bulb of *IPS-1* ^*−/−*^ mice 4 dpi. WT and IPS-1 deficient mice were infected intraperitoneal with LGTV and olfactory bulb, cerebrum, cerebellum and brain stem was collected 4 dpi. LGTV RNA levels were quantified with the NS3 real-time qPCR assay (detection limit 10 copies). Asterisks indicates statistical significance between *IPS-1* ^*−/−*^ olfactory bulb compared to other brain regions and calculated by Mann-Whitney test. Number sign indicates not detectable. (PDF 45.6 KB)
