# Supplementary figures and images for: Type I Interferon response in olfactory bulb, the site of tick-borne flavivirus accumulation, is primarily regulated by IPS-1
Source: J Neuroinflammation. 2016 Jan 27;13:22. doi: 10.1186/s12974-016-0487-9 (PMC4730761; doi:10.1186/s12974-016-0487-9)

## Additional file 1: Figure S1

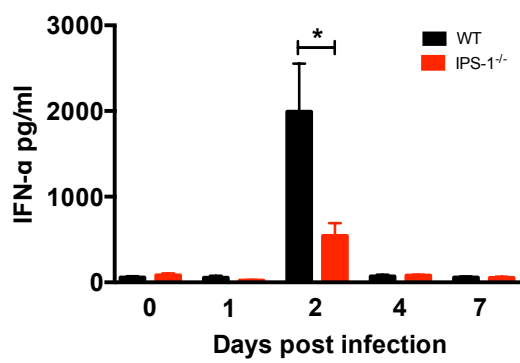

Supplement: Additional file 1: Figure S1. — IPS-1 −/− mice have lower IFN-α levels in serum after LGTV infection compared to WT mice. WT and IPS-1 −/− mice were infected intraperitoneally with 104 FFU of LGTV, and serum samples were collected at indicated time points (n = 5–10). The amount of IFN-α in the mouse serum was determined by enzyme linked immunosorbent assay (ELISA) according to the manufacturer’s instructions (PBL). Significance was calculated with student’s T test, *p < 0.05. (PDF 53.6 KB) [file 12974_2016_487_MOESM1_ESM.pdf]

## Additional file 2: Figure S2

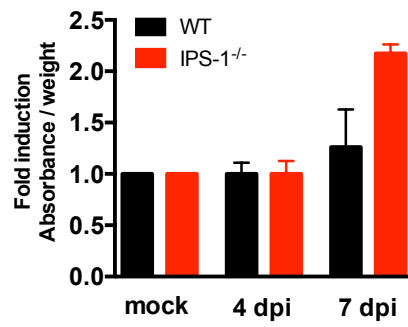

Supplement: Additional file 2: Figure S2. — Effect of IPS-1 signaling on BBB permeability upon LGTV infection. WT and IPS-1 −/− mice were mock or intraperitoneally infected with 104 FFU of LGTV (n = 3). Mice were intravenously injected with 100 μl 2 % Evans blue (Sigma) in PBS 4 and 7 dpi. After 1 h, animals were transcardially perfused with 20 ml PBS and the brains were removed. The brains were weighted and homogenized in 50 % TCA, and absorbance was measured at 610 nm. The absorbance was divided by the weight of the sample and normalized to mock infected samples. (PDF 42.5 KB) [file 12974_2016_487_MOESM2_ESM.pdf]

**Figure S3.**

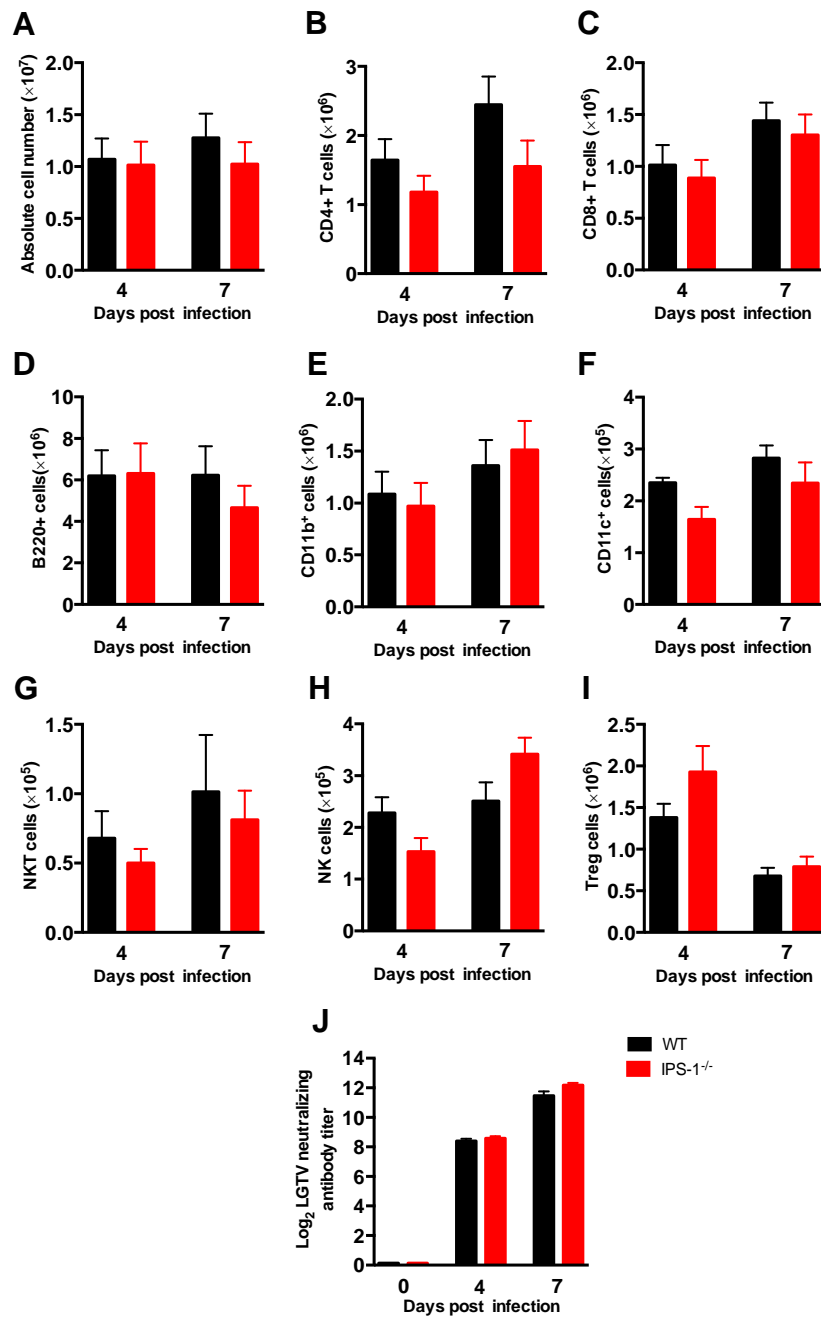

Supplement: Additional file 3: Figure S3. — IPS-1 does not influence the humoral response or cell composition or number in the spleen. WT and IPS-1 −/− mice were infected intraperitoneally with 104 FFU of LGTV. Spleens were harvested 4 and 7 dpi, and immune cells were isolated, counted, stained for various cell markers and analyzed by flow cytometry (n = 5–10). (A) Absolute cell numbers. (B) Total CD4+ T cells. (C) Total CD8+ cells. (D) Total B220+ cells. (E) Total CD11b+ cells. (F) Total CD11c+ cells. (G) Total NKT cells. (H) Total NK cells. (I) Total regulatory T (Treg) cells. (J) WT and IPS-1 −/− mice were mock or intraperitoneally infected with 104 FFU of LGTV, and the serum was isolated on 0, 4, and 7 dpi. Neutralizing antibody titers were determined by virus neutralization assay. Data represents mean with SEM of 5–10 mice in each group per time point. (PDF 20.6 KB) [file 12974_2016_487_MOESM3_ESM.pdf]

## Additional file 5: Figure S4

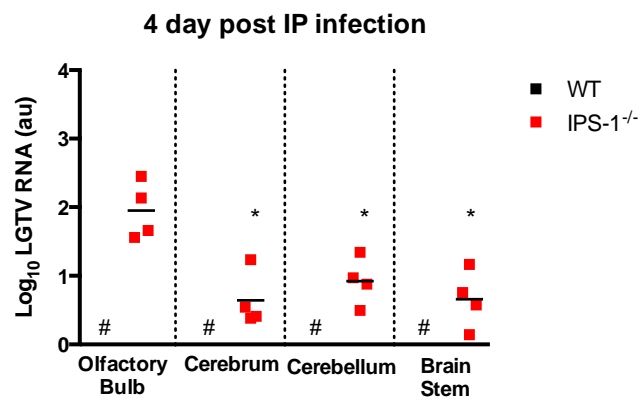

Supplement: Additional file 5: Figure S4. — Enhanced viral replication in olfactory bulb of IPS-1 −/− mice 4 dpi. WT and IPS-1 deficient mice were infected intraperitoneal with LGTV and olfactory bulb, cerebrum, cerebellum and brain stem was collected 4 dpi. LGTV RNA levels were quantified with the NS3 real-time qPCR assay (detection limit 10 copies). Asterisks indicates statistical significance between IPS-1 −/− olfactory bulb compared to other brain regions and calculated by Mann-Whitney test. Number sign indicates not detectable. (PDF 45.6 KB) [file 12974_2016_487_MOESM5_ESM.pdf]
