# Supplementary material for: Type I Interferon response in olfactory bulb, the site of tick-borne flavivirus accumulation, is primarily regulated by IPS-1
Source: J Neuroinflammation. 2016 Jan 27;13:22. doi: 10.1186/s12974-016-0487-9 (PMC4730761; doi:10.1186/s12974-016-0487-9)
Supplement: Additional file 4: — Method S1. Virus neutralization assay. Neutralization titer of antibody solution was determined by modified method of plaque reduction neutralization test (PRNT) called as rapid fluorescent focus inhibition test (RFFIT) [68, 69]. Briefly, mice sera were diluted 1:10 in serum-free maintenance media and heat-inactivated. The sera were serially diluted and incubated with 50 FFU LGTV for 1 h, and focus forming assay was performed. Neutralizing antibody titer was calculated as the reciprocal of serum dilution that gave 50 % (NT50) reduction of the number of FFU as compared to virus control. The test was accepted if virus dose was in the range of 30–90 FFU. (DOC 21.5 KB) [file 12974_2016_487_MOESM4_ESM.docx]

**Additional file 4: Method S1**

**Virus neutralization assay**

Neutralization titer of antibody solution was determined by modified method of plaque reduction neutralization test (PRNT) called as rapid fluorescent focus inhibition test (RFFIT) (1, 2). Briefly mice sera were diluted 1:10 in serum free maintenance media and heat inactivated. Sera were serially diluted and incubated with 50 FFU LGTV for 1 hr and focus forming assay was performed. Neutralizing antibody titer was calculated as the reciprocal of serum dilution that gave 50% (NT_50_) reduction of the number of FFU as compared to virus control. The test was accepted if virus dose was in the range of 30-90 FFU.

1. Vene S, Haglund M, Vapalahti O, Lundkvist A. A rapid fluorescent focus inhibition test for detection of neutralizing antibodies to tick-borne encephalitis virus. Journal of virological methods. 1998;73(1):71-5.

2. Kostense S, Moore S, Companjen A, Bakker AB, Marissen WE, von Eyben R, et al. Validation of the rapid fluorescent focus inhibition test for rabies virus-neutralizing antibodies in clinical samples. Antimicrob Agents Chemother. 2012;56(7):3524-30.
